# Angioid Streaks and Choroidal Neovascular Membrane Secondary to Pseudoxanthoma Elasticum: A Case Report

**DOI:** 10.7759/cureus.58104

**Published:** 2024-04-12

**Authors:** Liam Redden, Anuradha Mishra

**Affiliations:** 1 Ophthalmology, Dalhousie University, Halifax, CAN

**Keywords:** ocular complications, connective tissue disease, multidisciplinary approach, choroidal neovascular membrane, pseudoxanthoma elasticum, angioid streaks

## Abstract

Angioid streaks (AS) are recognized as irregular, linear dehiscences of Bruch’s membrane, often associated with systemic diseases. We present the case of a 50-year-old woman initially diagnosed with AS during a routine optometric examination. Subsequent ophthalmological evaluation revealed bilateral AS with calcified drusen. Two years post-diagnosis, she developed blurred vision in her right eye due to the choroidal neovascular membrane adjacent to the macular AS. Further evaluation uncovered clinical signs consistent with pseudoxanthoma elasticum (PXE), including characteristic skin lesions. A multidisciplinary approach involving ophthalmology, dermatology, and cardiovascular specialists was initiated. Histopathological confirmation of PXE was obtained through a skin biopsy. PXE, an autosomal recessive disorder characterized by elastin calcification, presents systemic manifestations necessitating comprehensive evaluation and monitoring. This case demonstrates the importance of recognizing ocular complications in PXE and advocates for early multidisciplinary intervention to mitigate potential vision and life-threatening outcomes.

## Introduction

Angioid streaks (AS) are irregular, linear, dark reddish-brown, crack-like dehiscences of Bruch’s membrane (a thin connective tissue that separates the retinal pigment epithelium from the choroid) that emanate in a radiating fashion from the optic nerve [1]. They can be idiopathic, but importantly, over 50% can be associated with systemic diseases such as pseudoxanthoma elasticum (PXE), Ehlers-Danlos syndrome, Paget disease, hemoglobinopathies, and other connective tissue diseases [1]. We present the case of a 50-year-old woman initially diagnosed with AS during a routine optometric examination who then went on to lose vision and be diagnosed with PXE.

## Case presentation

**Figure 1:**
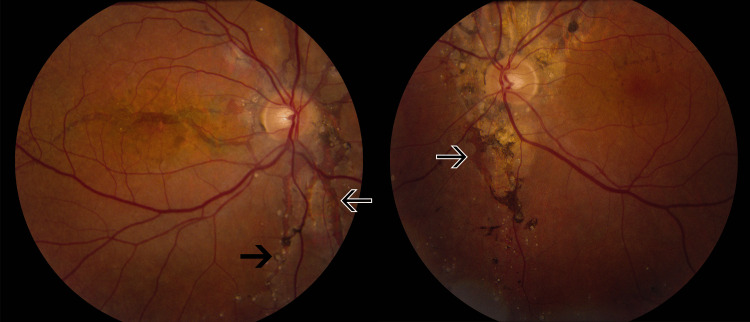
Color fundus photography of the right (two arrows) and left eyes (one arrow) Prominent AS (black arrows with white borders) are present, bilaterally radiating around the optic nerve, and can be particularly appreciated as a faint, dark red streak passing through the right eye macula. Numerous calcified drusens (black arrows) are present in both eyes, along with some pigmentary changes. AS, angioid streaks

A 50-year-old woman presented to her optometrist for a routine examination. AS were noted bilaterally, and she was referred to ophthalmology. She was otherwise healthy, and her past medical and ocular history was unremarkable. Visual acuity was 20/20 and 20/30 in the right and left eyes, respectively. Retinal examination revealed prominent AS bilaterally with calcified drusen (Figure 1).

Two years following the initial ophthalmic exam, the patient experienced blurred vision in the right eye, an inability to read a visual acuity chart, and could only count fingers close to her face. This was secondary to the choroidal neovascular membrane (CNVM) that developed adjacent to the AS in the macula. CNVM is a severe complication of AS and other more common conditions, such as macular degeneration. Treatment was initiated in the form of intraocular anti-vascular endothelial growth factor (VEGF) injections with a retinal specialist.

**Figure 2:**
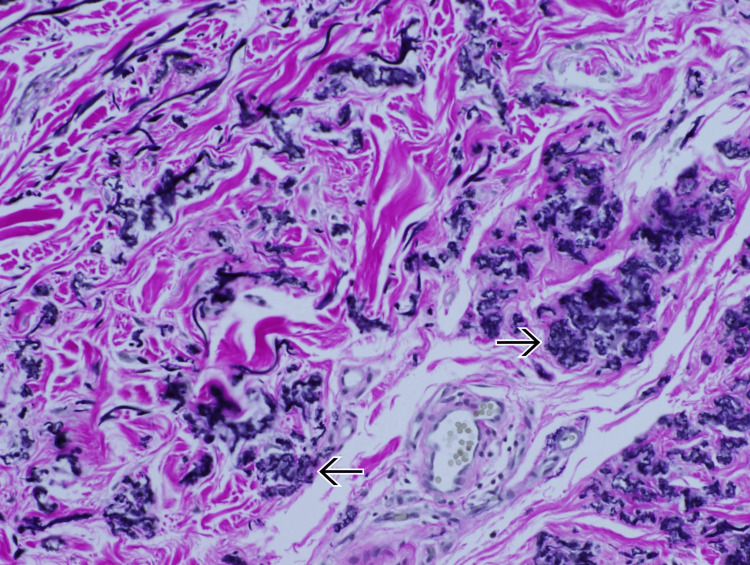
Histopathological slide of dermis retrieved from a punch biopsy of the posterior neck An elastic stain and a von Kossa stain demonstrate altered elastic fibers, showing fragmentation and clumping within the middermis. Fibers are basophilic due to calcium deposition. They can be appreciated as short, curled elastic fibers staining black within the dermis (black arrows).

A referral was made to the connective tissue disease clinic and dermatology. Skin examination found many grouped fleshy yellow to skin-colored papules in both the axillae and the posterior neck, with subtle redundant skin folds of the axilla and inguinal area. A punch biopsy of one of the neck lesions confirmed the diagnosis of PXE (Figure 2).

The patient had cardiac investigations, including an exercise stress test, echo, and CT coronary angiography, which were all unremarkable. Arrangements were made for her to be monitored every five years with repeat echo and CT scans to evaluate for aortic aneurysms.

## Discussion

PXE is an uncommon, autosomal recessive disease in which dystrophic calcification of elastin results in cardiovascular, ocular, cutaneous, and other complications [2]. Of the connective tissue diseases, PXE is the one most commonly associated with AS [3]. There is a high prevalence of peripheral artery disease, as well as impairment of left ventricular diastolic function and of the elastic properties of the aorta [4]. As outcomes can be potentially life-threatening (myocardial infarction and ischemic stroke) and/or vision-threatening, early referral to the appropriate specialists is suggested for evaluation and monitoring. There is no cure for PXE; therefore, current treatments focus on symptomatic management, including anti-VEGF therapy (for ophthalmic manifestations), lifestyle and lipid reduction (for managing vascular risk factors), and surgical intervention (for severe cardiac manifestations) [2].

## Conclusions

This case report demonstrates a striking example of the classic findings of AS and is a reminder to cardiologists or dermatologists of potential serious ocular involvement in their PXE patients. A multidisciplinary approach is key to monitoring the systemic effects of this disease.
